# Hodgkin lymphoma: hypodense lesions in mediastinal masses

**DOI:** 10.1038/s41598-024-64253-8

**Published:** 2024-06-25

**Authors:** Adrian Damek, Lars Kurch, Friedrich Christian Franke, Andishe Attarbaschi, Auke Beishuizen, Michaela Cepelova, Francesco Ceppi, Stephen Daw, Karin Dieckmann, Ana Fernández-Teijeiro, Tobias Feuchtinger, Jamie E. Flerlage, Alexander Fosså, Thomas W. Georgi, Dirk Hasenclever, Andrea Hraskova, Jonas Karlen, Tomasz Klekawka, Regine Kluge, Dieter Körholz, Judith Landman-Parker, Thierry Leblanc, Christine Mauz-Körholz, Markus Metzler, Jane Pears, Jonas Steglich, Anne Uyttebroeck, Dirk Vordermark, William Hamish Wallace, Walter Alexander Wohlgemuth, Dietrich Stoevesandt

**Affiliations:** 1 Department of Radiology, University Hospital Halle/Saale, Ernst-Grube-Strasse 40, 06120 Halle/Saale, Germany; 2 Department of Nuclear Medicine, University of Leipzig, Leipzig, Germany; 3 Department of Pediatric Hematology and Oncology, St. Anna Children’s Hospital, Medical University of Vienna, Vienna, Austria; 4 St. Anna Children’s Cancer Research Institute, Vienna, Austria; 5 Erasmus MC-Sophia Children’s Hospital, Rotterdam, The Netherlands; 6 Princess Màxima Center for Pediatric Oncology, Utrecht, The Netherlands; 7 Department of Pediatric Hematology and Oncology, University Hospital Motol and Second Medical Faculty of Charles University, Prague, Czech Republic; 8 Division of Pediatrics, Department of Woman-Mother-Child, Pediatric Hematology-Oncology Unit, University Hospital of Lausanne, Lausanne, Switzerland; 9 Department of Pediatric Hematology and Oncology, University College London Hospitals, London, UK; 10 Department of Radiation Oncology, University Hospital Vienna, Vienna, Austria; 11 Pediatric Onco-Hematology Unit, Hospital Universitario Virgen Macarena, Sevilla, Spain; 12 Pediatric Hematology, Oncology and Stem Cell Transplantation, Dr. Von Hauner University Children’s Hospital Ludwig-Maximilians-University, Munich, Germany; 13 Department of Oncology, St. Jude Children’s Research Hospital, Memphis, TN USA; 14 Department of Medical Oncology and Radiotherapy, Oslo University Hospital, Oslo, Norway; 15 Institute of Medical Informatics, Statistics and Epidemiology (IMISE), University of Leipzig, Leipzig, Germany; 16 Department of Pediatric Hematology and Oncology, University Children’s Hospital, Bratislava, Slovakia; 17 Karolinska University Hospital, Astrid Lindgrens Childrens Hospital, Stockholm, Sweden; 18 Institute of Pediatrics, Jagiellonian University Medical College, Krakow, Poland; 19 Department of Pediatric Hematology and Oncology, Justus-Liebig University, Gießen, Germany; 20 Hôpital Armand-Trousseau Sorbonne Universitè, Paris, France; 21 Service d’Hématologie Pédiatrique, Hôpital Robert-Debré, Paris, France; 22 Pediatric Oncology and Hematology, Department of Pediatrics and Adolescent Medicine, University Hospital, Erlangen, Germany; 23 Department of Pediatric Hematology and Oncology, Our Lady’s Children’s Hospital, Dublin, Ireland; 24 Department of Pediatric Hematology and Oncology, University Hospitals Leuven, Leuven, Belgium; 25 Department of Radiation Oncology, Medical Faculty of the Martin-Luther-University, Halle (Saale), Germany; 26 Department of Paediatric Oncology, Royal Hospital for Sick Children, University of Edinburgh, Edinburgh, UK

**Keywords:** Thymic cyst, Hypodense lesion, Hodgkin lymphoma, Tomography, X-ray computed, Cancer, Cancer imaging, Outcomes research

## Abstract

Hypodense volumes (HDV) in mediastinal masses can be visualized in a computed tomography scan in Hodgkin lymphoma. We analyzed staging CT scans of 1178 patients with mediastinal involvement from the EuroNet-PHL-C1 trial and explored correlations of HDV with patient characteristics, mediastinal tumor volume and progression-free survival. HDV occurred in 350 of 1178 patients (29.7%), typically in larger mediastinal volumes. There were different patterns in appearance with single lesions found in 243 patients (69.4%), multiple lesions in 107 patients (30.6%). Well delineated lesions were found in 248 cases (70.1%), diffuse lesions were seen in 102 cases (29.1%). Clinically, B symptoms occurred more often in patients with HDV (47.7% compared to 35.0% without HDV (p = 0.039)) and patients with HDV tended to be in higher risk groups. Inadequate overall early-^18^F-FDG-PET-response was strongly correlated with the occurrence of hypodense lesions (p < 0.001). Patients with total HDV > 40 ml (n = 80) had a 5 year PFS of 79.6% compared to 89.7% (p = 0.01) in patients with HDV < 40 ml or no HDV. This difference in PFS is not caused by treatment group alone. HDV is a common phenomenon in HL with mediastinal involvement.

## Introduction

Hodgkin lymphoma (HL) in childhood and adolescence is among the cancer entities with the highest 5 year survival rates, currently exceeding 90%^1–3^.

Long-term effects caused by tumor therapy limit the quality of life^4,5^ and survival in HL. Solid tumors are the main cause (other than HL itself) of death for long time survivors^6^. Relative risk of a coronary heart disease after mediastinal radiotherapy is also increased^7^. Therefore a lot of effort was directed towards decreasing side effects by avoiding radiotherapy^8^. In consequence, prognostic markers are needed to identify high risk patients in order to modify their treatment.

Hypodense volumes (HDV) in the thymus on contrast enhanced (CE) CT-scans were previously described as necrosis in mediastinal HL only in relatively small patient groups^9,10^. In 1990 Hopper et al. found no statistically significant correlation between the occurrence of hypodense lesions and progression free survival (PFS)^9^. In T-cell lymphomas hypodense lesions predicted a worse outcome^11^. Necrosis can be associated with hypoxia which is an adverse prognostic factor in solid tumors, as it is associated with tumor progression and resistance to therapy^12,13^.

Tumor volume is an established prognostic marker in HL. Tumor volume can be estimated as an ellipsoid (calculated by multiplying the horizontal length (a) with horizontal length (b) and vertical length (c) divided by two at initial staging)^14,15^.

In the EuroNet-PHL-C1 trial therapy was adjusted to tumor volume, B-symptoms and therapy response. Patients of Ann-Arbor stages I A/B and II A were assigned to therapy group-1 (TG1), stages IA/B_E_, IIA_E_, II B or III A to TG2 and stages IIB_E_, IIIA/B_E_, III B or IV A/B to TG3. All therapy groups received two intensive induction cycles (vincristine, etoposide, prednisone and doxorubicin; OEPA scheme). TG2 and TG3 received two or four additional cycles. These TG2 and TG3 patients were randomly assigned to a COPDAC (cyclophosphamide, vincristine, prednisone and dacarbazine) or COPP (cyclophosphamide, vincristine, prednisone, procarbazine) scheme to reduce gonadotoxicity. All patients received early response assessment (ERA) with a positron emission tomography (PET)/CT-scan after two cycles of chemotherapy. Early interim fluorodeoxyglucose (^18^F-FDG) PET-positivity after two cycles of chemotherapy strongly correlated with a poor PFS^16^. Therefore only ^18^F-FDG-PET-positive patients received radiotherapy in order to minimize long-term effects. Due to this individualized therapy there was a homogenization of PFS around 80–95% in all therapy groups^3,17^.

### Objective

The aim of this study was to describe the incidence, factors contributing to the occurrence and characteristics of HDV as well as its possible value as a prognostic marker in a large group of CHL patients enrolled in the EuroNet-PHL-C1 trial.

## Materials and methods

This study was based on images and clinical data of the prospective EuroNet-PHL-C1 trial (EudraCT: 2006-000995-33; Clinicaltrial.gov: NCT00433459) which recruited 2102 pediatric and adolescent patients with CHL between 2007-30-01 and 2013-29-01^3^. Original imaging data (18F-FDG-PET, CT, MRI) of 1752 patients was available for central review.

According to national legislation, the EuroNet-PHL-C1 trial was approved by ethics committees, medical agencies, and institutional review boards of the participating countries and centers. All patients and/or their guardians gave written informed consent. The institutional review board of the EuroNet-PHL-C1 trial approved this retrospective imaging data analysis and waived the requirement for additional informed consent. The study was performed in accordance with good clinical practice and the Declaration of Helsinki. The datasets generated during and/or analysed during the current study are available from the corresponding author on reasonable request.

**Figure 1:**
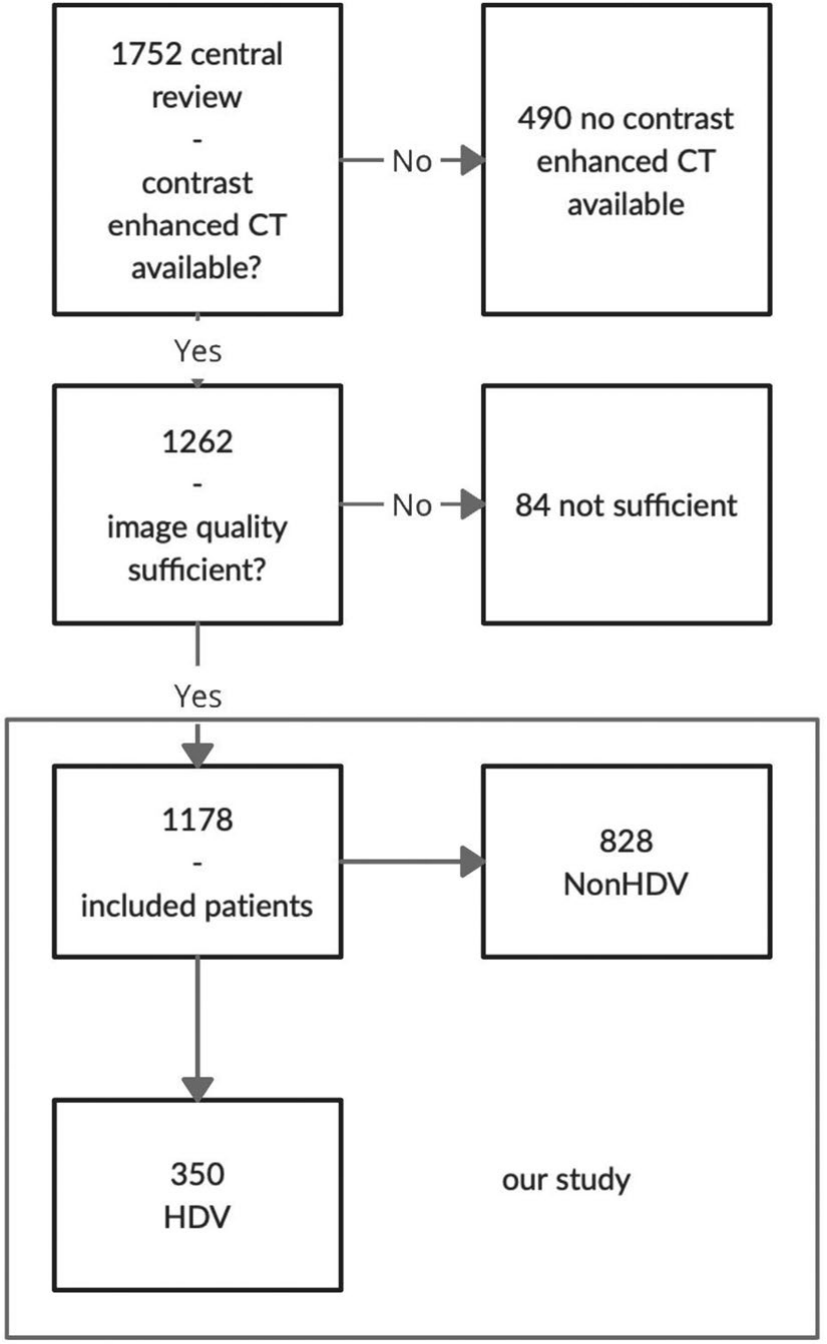
Flowchart of patient inclusion and patient groups in the study.

A total of 1262 patients who received a CE CT scan on initial staging and had mediastinal lymphoma involvement were eligible for our study. We excluded 84 patients because of technical reasons like missing or incomplete DICOM-data, insufficient image quality (defined as: slice thickness of > 5 mm, incomplete imaging of the tumor, motion- ring- or beam-hardening-artifacts, low image resolution). Our final study population comprised 1178 patients (see Fig. 1).

The following parameters were recorded in the C1-database: age at registration, sex and occurrence of B symptoms (fever, drenching night sweats and weight loss), hemoglobin, serum albumin and erythrocyte sedimentation rate. The initial mediastinal tumor volume was calculated as a*b*c/2 (TV_abc_). Correspondingly the volume on ERA was assessed (TV_ERA_). Morphologic ERA-response was defined as (1-(TV_ERA_/TV_abc_)*100). Overall ^18^F-FDG-PET-positivity status on early response assessment was recorded as well as Ann-Arbor stages and TG. PFS status was also taken from the C1-data-base, median follow-up was 64 months.

Reading was preferably done on CT scans with a medium smooth reconstruction kernel and a slice thickness of 5 mm. This was done in only 49.4% of all 1178 cases. In the remaining cases, recalculated data from slice thicknesses between 0.5 and 4.0 mm were used. The computed tomography dose index (CTDI)vol strongly varied due to the multinational multicenter design of the study with patients from 152 study sites and was not documented in all cases. In the CT images used for this publication, the CDTIvol varied from 2.52 to 8.64 mGy.

**Figure 2:**
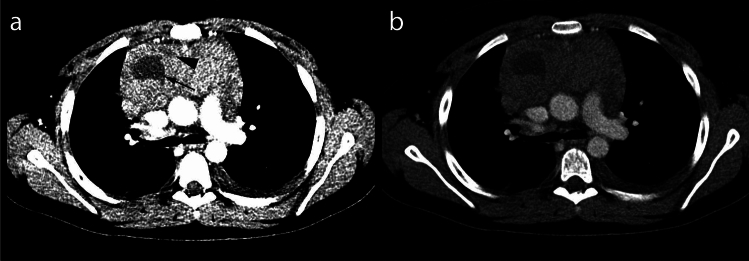
Hypodense lesion evaluation presets. (**a**) WL: 34 WW: 88, second hypodense lesion (arrowhead) ventromedial of the better visible larger lesion (arrow). (**b**) WL: 100 WW: 300, ventral lesion less visible. The hypodense lesions in this case have an average density of 21 HU, the rest of the tumor of 57 HU (CTDI 4.52 mGy).

Standardized preset values for observing the CT images were applied. For tumor measurements window level (WL) 50 Hounsfield units (HU) and window width (WW) 350 HU was chosen. For measurement of hypodense volumes two narrow window settings were used (WL: 100 WW: 300 and WL: 34 WW: 88) (see Fig. 2).

HDV was defined as a clearly delineated area more than 30 HU below the surrounding tumor tissue attenuation. For measuring these were encircled in each slice and corresponding to Gossmann et al.^18^ measured as a volume (TV_3D_). For the measurement of the mediastinal tumor volume we compared the volumetric approach (TV_3D_) to the TV_abc_ method used in the C1-trial.

Hypodense lesions were visually classified as well delineated or diffuse (see Fig. 4). Diffuse HDV was defined as tumor margins that blended into the surrounding tissue without a clear demarcation. Osirix MD Software by pixmeo (version 5.5-10) was used for measurements. All data analyses were performed using R version 4.0.2.

## Results

There was no significant difference in sex (p = 0.54) or Ann Arbor stages (p = 0.457) between patients included in our subanalysis and the non-included C1 trial population, however there was a slight increase in patients older than 13 years in the included group (p = 0.0182).

**Figure 3:**
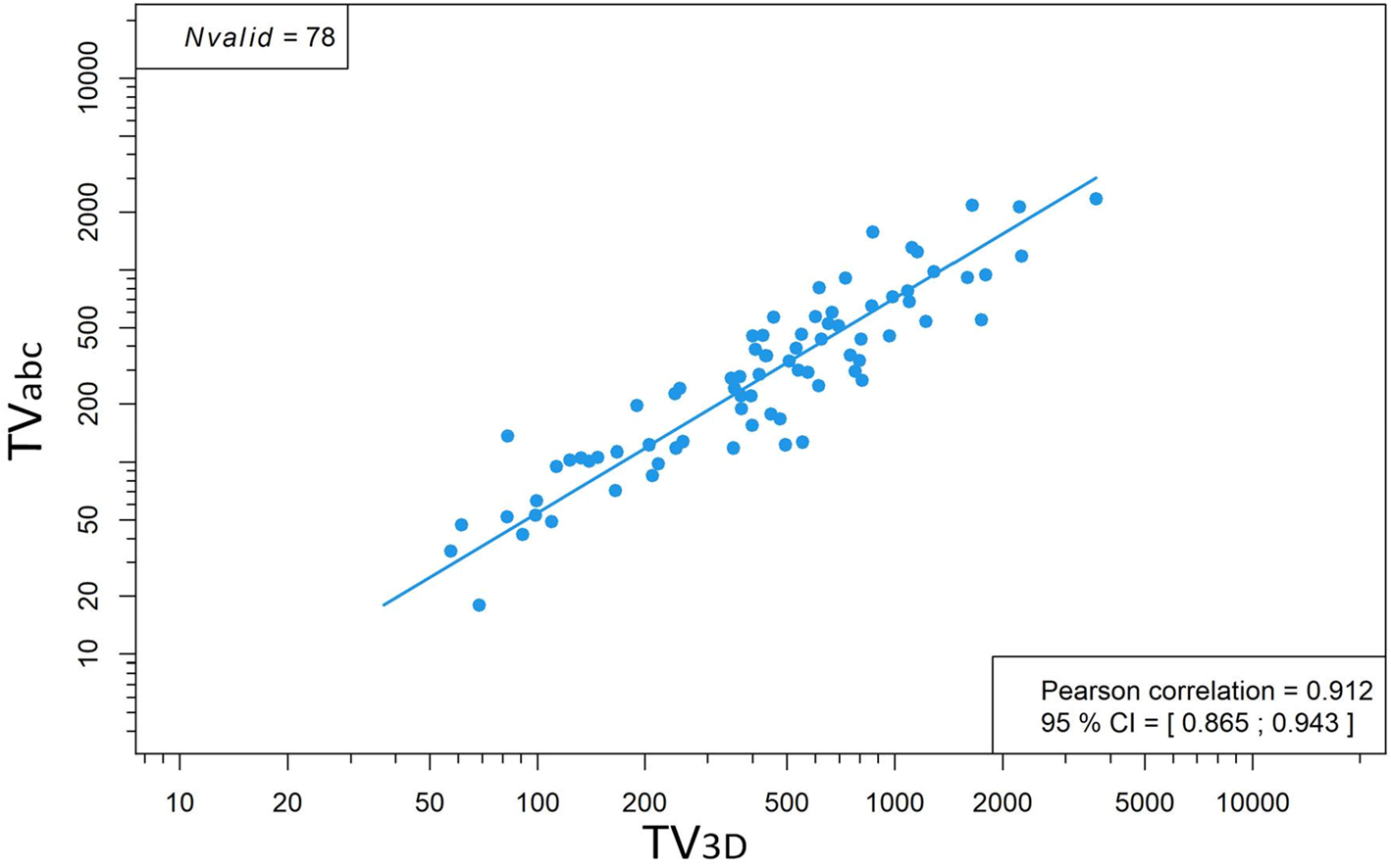
Strong correlation between TV_3D_ and TV_abc_ assessment of mediastinal volumes.

We performed the TV_abc_ and the TV_3D_ measurement in all patients with more than 10% of HDV in the tumor and found the latter to be time consuming (45–75 min per patient). A Pearson correlation of mediastinal TV3D and mediastinal TV_abc_ was 0.912 (95% CI [0.865; 0.943]) (see Fig. 3). The TV_3D_ measurement is too time consuming in a routine clinical setting and the accurate TV_abc_ method was solely used for patients with smaller HDV.

Therefore most of the other calculations were performed using mediastinal TV_abc_, because the method can be considered as valid. The hypodense ratio was defined as HDV divided by mediastinal TV_abc_.

**Figure 4:**
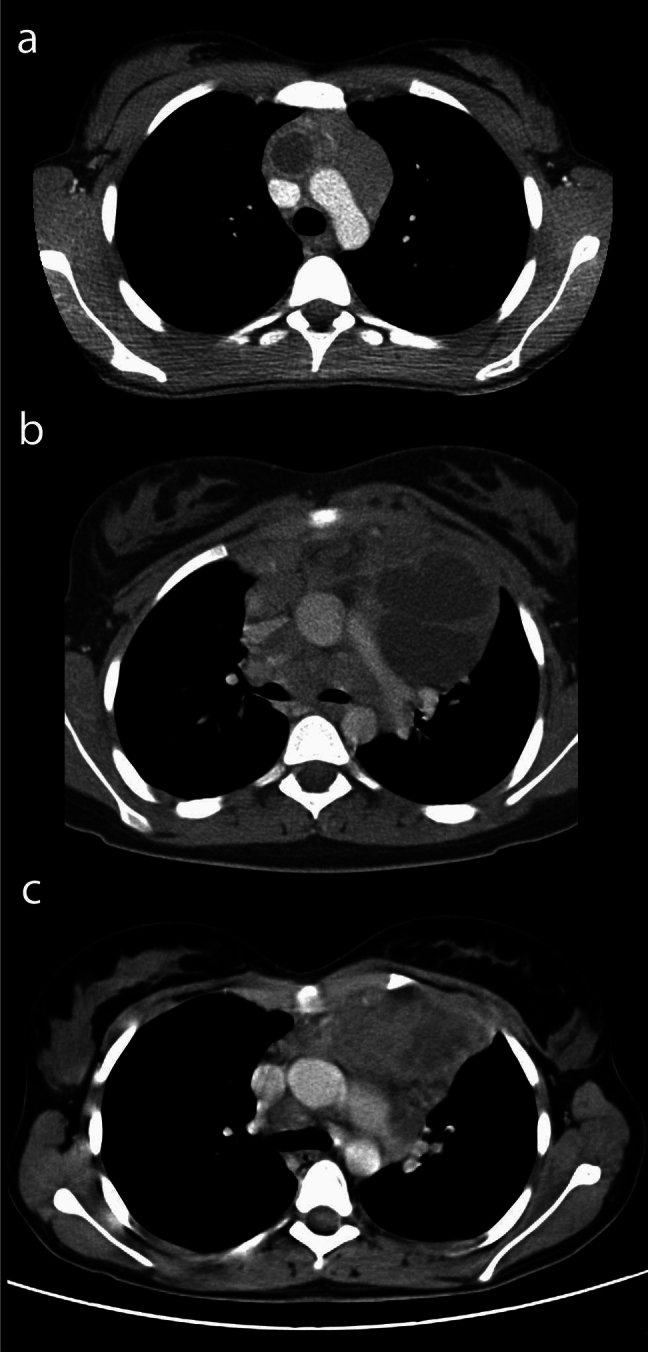
Variety of appearances of hypodense lesions. (**a**) Single lesion with enhancing wall (CTDI 2.52 mGy, average lesion density 29 HU), (**b**) multiple sharply delineated lesions (CTDI 6.56 mGy average lesion density 16 HU), (**c**) diffuse lesions (CTDI 8.64 mGy average lesion density 29 HU).

1178 patients were included in this study. 350 patients (29.7%) had at least one HDV. There were different patterns in appearance. Single lesions were found in 243 patients (69.4%), multiple lesions in 107 patients (30.6%). Well delineated lesions were more common and found in 248 cases (70.1%) while diffuse lesions were seen in 102 cases (29.1%) (see Fig. 4).

HDV typically occurred in larger mediastinal volumes. Mediastinal TV_3D_ and HDV correlated strongly (Pearson correlation was 0.908; 95% CI [0.859; 0.941]). Mediastinal TV_abc_ also correlated with the HDV (Pearson correlation was 0.503; 95% CI [0.419; 0.578]).

B symptoms occurred in patients with HDV in 47.7% and in 35.0% of patients without HDV (Chi-Square-test, p = 0.039). If B symptoms were observed separately there was a significant difference between the HDV and non-HDV group (fever: p = 0.018; night sweats: p = 0.001; weight loss p < 0.001). HDV were not correlated to inflammatory parameters like serum albumin, hemoglobin, or erythrocyte sedimentation rate.

Patients with HDV were statistically significantly detectable in higher therapy groups (TG1: 27.4% vs. 40.1%, TG2: 20.3% vs. 20.8%, TG3: 52.3% vs. 39.1%; p = 0.021).

If corrected for B symptoms, other prognostic factors from the C1-trial (erythrocyte sedimentation rate, serum albumin and hemoglobin) partially correlated with mediastinal TV_abc_ but not with HDV.

Overall ERA-^18^F-FDG-PET-response correlated with the occurrence of HDV (p-value < 0.001).

Patients with larger HDV showed slightly higher incidence of overall ERA-^18^F-FDG-PET-positivity in a binomial regression (p < 0.01). There was no correlation between the ERA volume response and HDV (HDV 81.3% vs. 80.8%; p = 0.88).

The presence of HDV was not significantly correlated to PFS (LogRank-test, p = 0.481).

In a next step the group of patients with a hypodense ratio ≥ 10% was defined as the high hypodense ratio and the rest as low hypodense ratio group. The high hypodense ratio group consisted of 78 (6.6%) patients. In this high hypodense group the ratio from 10 to ≤ 20% was present in 51 (65.4%) patients, between > 20% and ≤ 30% in 10 (12.8%), between > 30% and ≤ 40% in 11 (14%), between > 40% and ≤ 50% in 4 (5.1%), between > 50% and ≤ 60% in 1 (1.3%) and between > 60% and ≤ 70% in 1 (1.3%). There were no patients with a hypodense ratio of more than 70%.

The hypodense ratio did not correlate with mediastinal TV_abc_ (Pearson correlation was 0.075 (95% CI [− 0.031, 0.180]).

There was also no significant correlation between high hypodense ratio and mediastinal TV_3D_, a Pearson correlation only showed a weak effect size with a coefficient of − 0.031 (95% CI [− 0.252, 0.193]).

There was no correlation between the high hypodense ratio and PFS (89.1% in the low and 87.2% in the high ratio group; p = 0.739).

In a third step the significance of large HDVs was analyzed.

**Figure 5:**
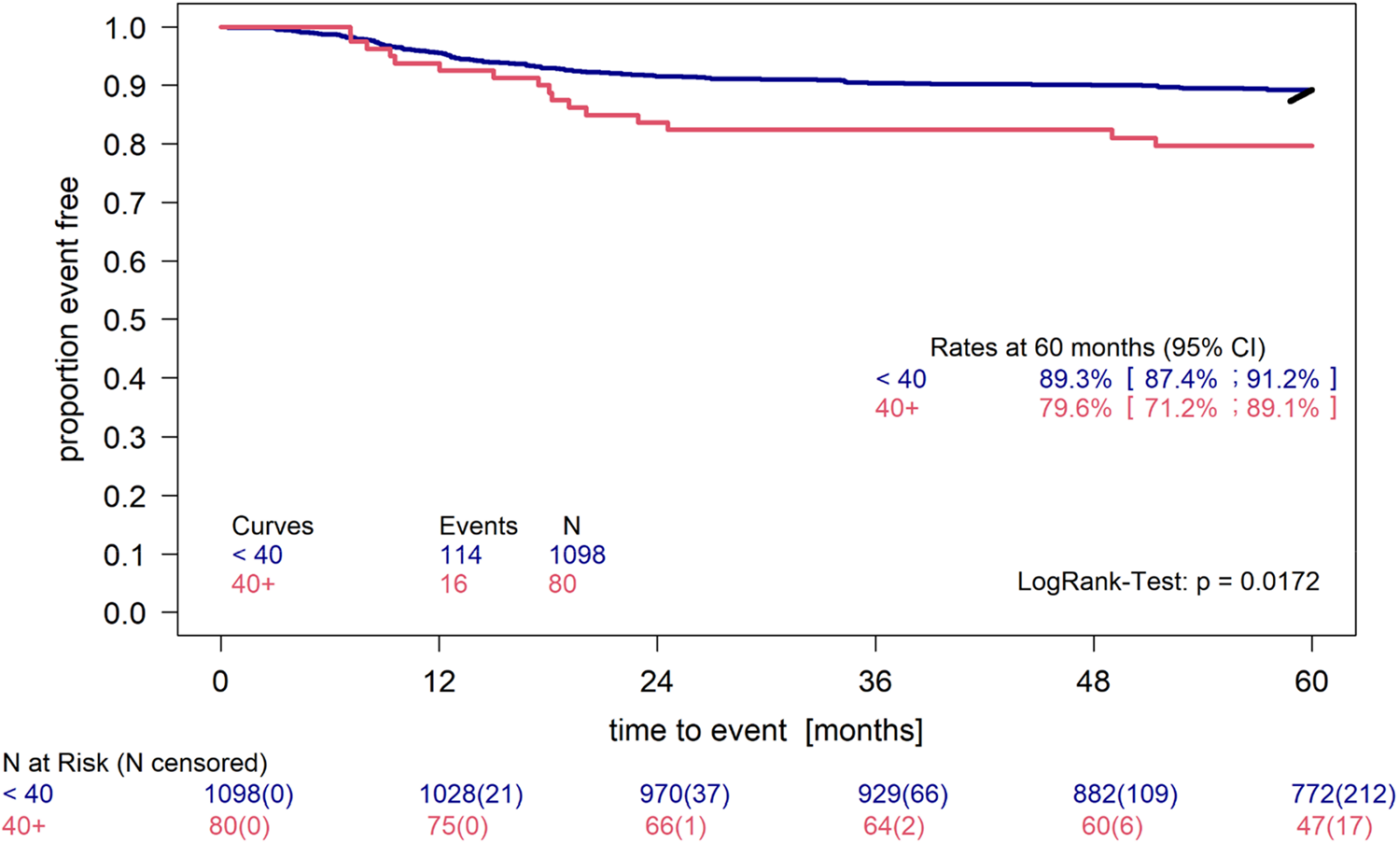
Kaplan–Meier plot of the HDV > 40 ml group. Patients with volumes above 40 ml (n = 80) compared to all other patients with sufficient PFS data (n = 1098). LogRank Test p = 0.0172.

Thus small volumes may be common and may have no influence on PFS, a cut-off value of 40 ml, roughly corresponding to the mean value plus one standard deviation on the log scale, was chosen. The group with volumes larger than 40 ml (n = 80) had a 5 year PFS of 79.6% (95% CI [71.2%; 89.1%]) instead of 89.3% (95% CI [87.4%; 91.2%]). This difference was significant (p = 0.017) (see Fig. 5).

**Figure 6:**
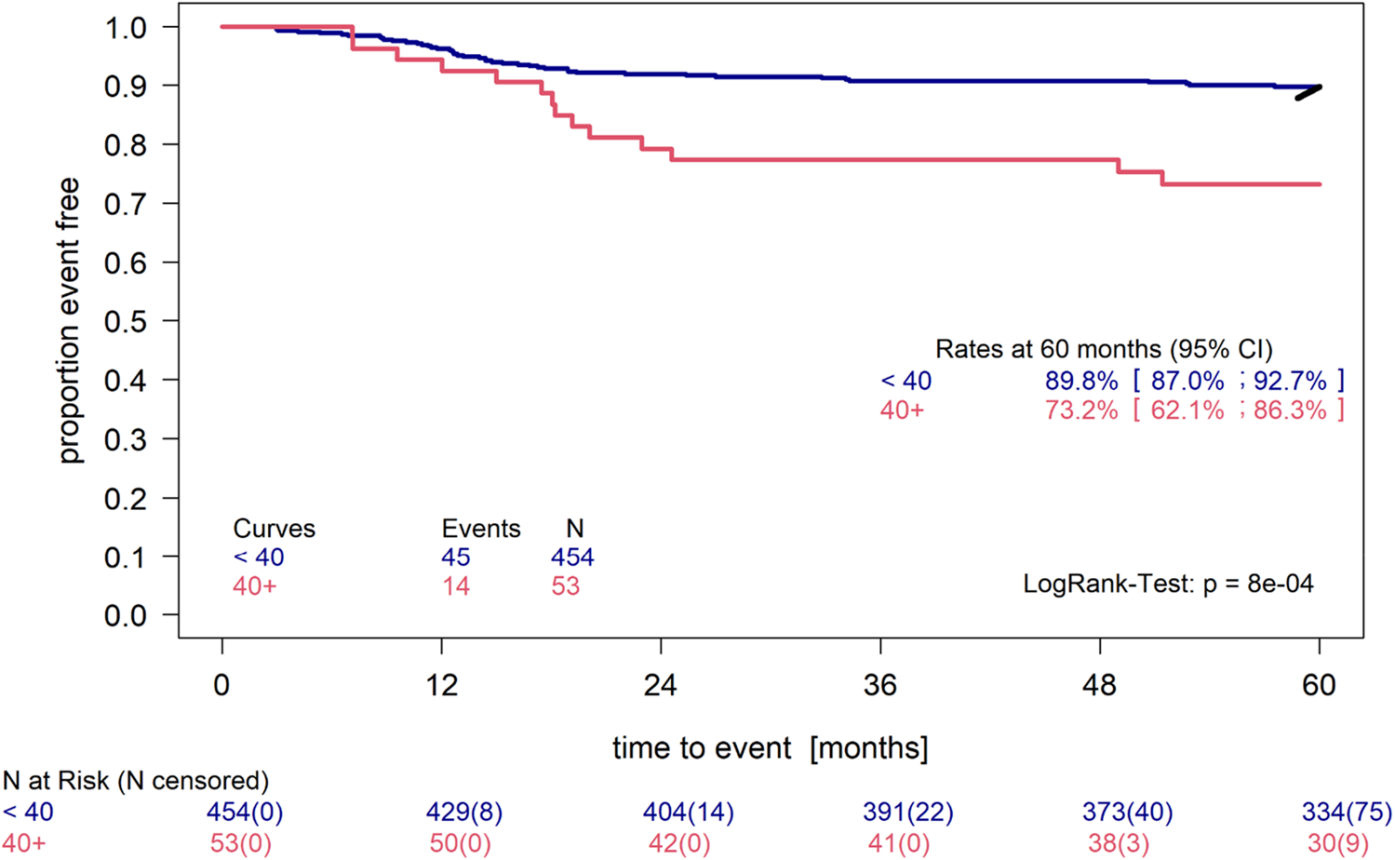
Kaplan–Meier plot of the HDV > 40 ml group in TG3 compared to all patients in TG3 with HDV < 40 ml. Patients with volumes above 40 ml (n = 53) compared to all other patients with sufficient PFS data (n = 454). LogRank test p = 0.0008.

The size of this difference is robust when using 60 ml or 100 ml as cut-off. The 80 patients having 40 ml HDV or more were distributed to TGs as follows (16.2% TG1, 17.5% TG2, 66.2% TG3). 14 out of 16 relapses were from the TG3 group. In a subgroup analysis of only TG3 patients the difference in PFS persisted (see Fig. 6.) and was even more pronounced (p = 0.0008).

## Discussion/conclusion

Hopper et al.^9^ concluded that hypodense areas do not have an impact on PFS. In contrast, in our study larger HDV (> 40 ml) showed an impact on PFS. It has been postulated by Hopper et al. that HDV is the correlate of necrosis^9^. Also hypoxia is associated with tumor progression and resistance to therapy^12,13^. Necrosis and the resulting hypoxia would be a possible pathophysiological explanation for the negative impact of large HDV on patient prognosis.

Patients with HDV > 40 ml are enriched in advanced stages but the subgroup analysis of TG3 patients shows a persistent difference in PFS (p = 0.0008). Indicating that the difference in PFS is not caused by TG alone. Further research is needed to clarify whether HDV is an epiphenomenon in larger tumors or an independent prognostic indicator for lower PFS. Patients with HDV may require increased follow-up in future studies. The effects of radiotherapy on HDV also need to be further investigated.

The a*b*c/2-method as a surrogate 3D-measurement of volumes has been established for different settings^14^ and seems less time consuming than true 3D-measurements^19^. Since auto segmentation of mediastinal tumor volumes is more complicated than lung nodule volumetry^20^ algorithms for automated detection could not be used in our study. Therefore mediastinal TV_abc_ has been used as the standard volume measurement method in EuroNet-PHL trials. The present sub-study showed that correlations of HDV and mediastinal TV_abc_ were not as strong as HDV and mediastinal TV_3D_. The TV_abc_ method assumes an ellipsoid mediastinal tumor shape, but this is not always reflecting true tumor shape. This may be due to limited mediastinal space, which influences tumor growth. Since the TV_3D_ method is extremely time consuming and not feasible for clinical practice, mediastinal TV_abc_ has also been used in this sub-study.

With increasing mediastinal tumor volume, there was a higher probability of HDV occurrence. If HDV was present, it strongly correlated with the mediastinal tumor volume. Therefore it can be postulated that their occurrence is a biological phenomenon in CHL. Hypodense lesions have been described in other tumor entities like nasopharyngeal carcinoma^21^. Yang et al. postulated that in NHL areas with reduced or missing contrast enhancement should be considered to represent intratumoral necrosis. This, as well as inhomogeneous density, proved to be a negative predictor for a “good outcome” defined as patients that survived for more than 24 months with no evidence of recurrence^11^.

Hopper et al. first described HDV in HL in 1990. They also considered low-attenuated areas on CT scans with an average of 10–27 HU to be nodal necrosis. In an adult HL population of 107 patients, 21% had necrosis of at least a portion of their nodal disease as shown by thoracic CT on initial staging^9^. Although it has been postulated by Hopper and Yang^9,11^ there are no larger studies that histologically correlate HDV to necrosis.

Overall ^18^F-FDG-PET-positivity on early interim staging is a unfavorable prognostic indicator with regard to PFS^16^. Therefore, this was used in the C1-trial to assign patients to radiotherapy after the end of chemotherapy. In this study ^18^F-FDG-PET response correlated with the presence and also the size of HDV. Therefore HDV might be a surrogate parameter for ^18^F-FDG-PET lymphoma treatment response where ^18^F-FDG-PET is not accessible.

In HL B symptoms correlate with poor PFS^22^. In our study the occurrence of hypodense lesions correlated with B symptoms but there was no correlation of HDV and PFS. The missing correlation could be explained by the fact that B symptoms led to an intensified therapy scheme in the C1-trial^3^.

Vecchi et al.^22^ and Lee et al.^23^ observed a worse therapy outcome for large mediastinal masses. Yang et al.^11^ showed a correlation between inhomogeneous density in the tumor pattern and a poor survival outcome in 51 NHL patients. Analogue changes in MRI associated with necrosis in nasopharyngeal carcinoma are an established prognostic factor^21^. HDV in HL and their correlation to PFS has rarely been surveyed. Hopper et al.^9^ investigated whether low-attenuation, complex cystic areas on CT scans in mediastinal nodal disease have an impact on remission and overall survival. Besides a difference in size (average diameter of the nodal mass: 9.5 cm tumor with necrotic part vs. 8.5 cm) and tumor volume (average volume of the nodal mass: 1274 cm^3^ tumor with necrotic part vs. 876 cm^3^), they found no difference between patients with HDV and without^9^. Our cohort consisted of 1178 pediatric and adolescent patients, whilst their cohort contained only 107 adult (18 years of age or older) patients and used a different scanning protocol (10 mm slices).

There are several limitations in our study. Due to its design as a multicenter study there was a wide range of different CT protocols with different contrast agent injection rates and doses. Low doses of contrast medium used may have caused some small HDVs to be missed. RSeveral patients with Non-CE-CT had to be excluded, although hypodense lesions were present. The threshold for the presence of small HDV was hard to define, because it was not always distinguishable from fatty tissue or trapped pericardial effusion. Since patients with HDV were more likely to receive intensified treatment and radiotherapy the prognostic value of HDV was possibly leveled out and therefore underestimated in this study.

### Conclusion

Hypodense lesions are common in CHL, typically appearing in larger mediastinal masses. HDV are linked to higher stages of CHL and HDV larger than 40 ml are linked to a poor prognosis in children and adolescents. Further research with additional ^18^F-FDG-PET-data should be considered to clarify whether HDV is an epiphenomenon in larger tumors or an independent indicator for PFS.

## Data Availability

The datasets generated during and/or analysed during the current study are available from the corresponding author on reasonable request.

## Abbreviations

CE: Contrast enhanced
CHL: Classical Hodgkin lymphoma*
COPDAC: Cyclophosphamide, vincristine, prednisone and dacarbazine
COPP: Cyclophosphamide, vincristine, prednisone, procarbazine
CT: Computed tomography
CTDIvol: Computed tomography dose index in helical CT
DICOM: Digital imaging and communications in medicine
ERA: Early response assessment
EuroNet-PHL: European Network for Pediatric Hodgkin Lymphoma
18F-FDG: Fluorodeoxyglucose
HDV: Hypodense volumes
HL: Hodgkin lymphoma (including nodular type of lymphocyte predominant Hodgkin lymphoma and CHL)
HU: Hounsfield units
TV3D: Tumor volume measured slice by slice
TVabc: Tumor volume measured (a*b*c)/2
TVERA: Tumor volume at early response assessment
OEPA: Vincristine, etoposide, prednisone and Doxorubicin
PET: Positron emission tomography
PFS: Progression free survival
TG: Therapy group
WL: Window length
WW: Window width

## References

[1] Friedman DL, Chen L, Wolden S (2014). Dose-intensive response-based chemotherapy and radiation therapy for children and adolescents with newly diagnosed intermediate-risk Hodgkin lymphoma: A report from the children’s oncology group study AHOD0031. J. Clin. Oncol..

[2] Schellong G, Pötter R, Brämswig J (1999). High cure rates and reduced long-term toxicity in pediatric Hodgkin’s disease: The German–Austrian multicenter trial DAL-HD-90. The German–Austrian pediatric Hodgkin’s disease study group. J. Clin. Oncol..

[3] Mauz-Körholz C, Landman-Parker J, Balwierz W (2022). Response-adapted omission of radiotherapy and comparison of consolidation chemotherapy in children and adolescents with intermediate-stage and advanced-stage classical Hodgkin lymphoma (EuroNet-PHL-C1): A titration study with an open-label, embedded, multinational, non-inferiority, randomised controlled trial. Lancet Oncol..

[4] Brierley JD, Rathmell AJ, Gospodarowicz MK (1998). Late effects of treatment for early-stage Hodgkin’s disease. Br. J. Cancer.

[5] Ekstrand BC, Horning SJ (2002). Hodgkin’s disease. Blood Rev..

[6] Aleman BMP, van den Belt-Dusebout AW, Klokman WJ (2003). Long-term cause-specific mortality of patients treated for Hodgkin’s disease. J. Clin. Oncol..

[7] van Nimwegen FA, Schaapveld M, Cutter DJ (2016). Radiation dose-response relationship for risk of coronary heart disease in survivors of Hodgkin lymphoma. J. Clin. Oncol..

[8] Zubizarreta PA, Alfaro E, Guitter M (2017). Children and adolescent Hodgkin lymphoma in Argentina: Long-term results after combined ABVD and restricted radiotherapy. J. Pediatr. Hematol. Oncol..

[9] Hopper KD, Diehl LF, Cole BA (1990). The significance of necrotic mediastinal lymph nodes on CT in patients with newly diagnosed Hodgkin disease. Am. J. Roentgenol..

[10] Li Y, Yang Z, Guo Y (2007). Contrast-enhanced multislice CT features and predominant anatomic distribution of mediastinal malignant lymphoma. Sheng Wu Yi Xue Gong Cheng Xue Za Zhi.

[11] Yang W, Jiang S, Lin J, Li Y (2019). CT findings predict survival of patients with peripheral T cell lymphoma: A preliminary study. Radiol. Oncol..

[12] Shannon AM, Bouchier-Hayes DJ, Condron CM, Toomey D (2003). Tumour hypoxia, chemotherapeutic resistance and hypoxia-related therapies. Cancer Treat. Rev..

[13] Kewitz S, Kurch L, Volkmer I, Staege MS (2016). Stimulation of the hypoxia pathway modulates chemotherapy resistance in Hodgkin’s lymphoma cells. Tumour Biol..

[14] Kothari RU, Brott T, Broderick JP (1996). The ABCs of measuring intracerebral hemorrhage volumes. Stroke.

[15] Won S-Y, Zagorcic A, Dubinski D (2018). Excellent accuracy of ABC/2 volume formula compared to computer-assisted volumetric analysis of subdural hematomas. PLoS One.

[16] Gallamini A, Hutchings M, Rigacci L (2007). Early interim 2-[18F]fluoro-2-deoxy-d-glucose positron emission tomography is prognostically superior to international prognostic score in advanced-stage Hodgkin’s lymphoma: A report from a joint Italian–Danish study. J. Clin. Oncol..

[17] Mauz-Körholz C, Landman-Parker J, Fernández-Teijeiro A (2023). Response-adapted omission of radiotherapy in children and adolescents with early-stage classical Hodgkin lymphoma and an adequate response to vincristine, etoposide, prednisone, and doxorubicin (EuroNet-PHL-C1): A titration study. Lancet Oncol..

[18] Gossmann A, Eich HT, Engert A (2005). CT and MR imaging in Hodgkin’s disease—Present and future. Eur. J. Haematol..

[19] Sohaib SA, Turner B, Hanson JA (2000). CT assessment of tumour response to treatment: Comparison of linear, cross-sectional and volumetric measures of tumour size. Br. J. Radiol..

[20] Bartlett EC, Kemp SV, Rawal B, Devaraj A (2022). Defining growth in small pulmonary nodules using volumetry: Results from a “coffee-break” CT study and implications for current nodule management guidelines. Eur. Radiol..

[21] Lan M, Huang Y, Chen C-Y (2015). Prognostic value of cervical nodal necrosis in nasopharyngeal carcinoma: Analysis of 1800 patients with positive cervical nodal metastasis at MR imaging. Radiology.

[22] Vecchi V, Pileri S, Burnelli R (1993). Treatment of pediatric Hodgkin disease tailored to stage, mediastinal mass, and age. An Italian (AIEOP) multicenter study on 215 patients. Cancer.

[23] Lee CK, Bloomfield CD, Goldman AI, Levitt SH (1980). Prognostic significance of mediastinal involvement in Hodgkin’s disease treated with curative radiotherapy. Cancer.

